# Selective Predation of a Stalking Predator on Ungulate Prey

**DOI:** 10.1371/journal.pone.0158449

**Published:** 2016-08-22

**Authors:** Marco Heurich, Klara Zeis, Helmut Küchenhoff, Jörg Müller, Elisa Belotti, Luděk Bufka, Benno Woelfing

**Affiliations:** 1 Bavarian Forest National Park, Department of Conservation and Research, Freyunger Straße 2, 94481 Grafenau, Germany; 2 Chair of Wildlife Ecology and Management, Albert Ludwigs University of Freiburg, Tennenbacher Straße 4, 79106 Freiburg, Germany; 3 Ludwig Maximilian University, Munich, Department of Statistics, Statistical Consulting Unit, Ludwigstraße 33, 80539 Munich, Germany; 4 Faculty of Forestry and Wood Sciences, Czech University of Life Sciences Prague, Kamýcká 1176, CZ-16521 Prague 6, Czech Republic; 5 Department of Research and Nature Protection, Šumava National Park Administration, Sušická 399, CZ-34192 Kašperské Hory, Czech Republic; 6 Field Station Fabrikschleichach, Department of Animal Ecology and Tropical Biology, Biocenter, University of Würzburg, Rauhenebrach, Germany; Université de Sherbrooke, CANADA

## Abstract

Prey selection is a key factor shaping animal populations and evolutionary dynamics. An optimal forager should target prey that offers the highest benefits in terms of energy content at the lowest costs. Predators are therefore expected to select for prey of optimal size. Stalking predators do not pursue their prey long, which may lead to a more random choice of prey individuals. Due to difficulties in assessing the composition of available prey populations, data on prey selection of stalking carnivores are still scarce. We show how the stalking predator Eurasian lynx (*Lynx lynx*) selects prey individuals based on species identity, age, sex and individual behaviour. To address the difficulties in assessing prey population structure, we confirm inferred selection patterns by using two independent data sets: (1) data of 387 documented kills of radio-collared lynx were compared to the prey population structure retrieved from systematic camera trapping using Manly’s standardized selection ratio alpha and (2) data on 120 radio-collared roe deer were analysed using a Cox proportional hazards model. Among the larger red deer prey, lynx selected against adult males—the largest and potentially most dangerous prey individuals. In roe deer lynx preyed selectively on males and did not select for a specific age class. Activity during high risk periods reduced the risk of falling victim to a lynx attack. Our results suggest that the stalking predator lynx actively selects for size, while prey behaviour induces selection by encounter and stalking success rates.

## Introduction

Predation is a key factor in shaping animal populations through direct killing of prey [1] and by provoking costs for anti-predator responses [2]. Prey selection can dramatically affect the demography of prey populations [3], predator–prey community dynamics [4] and evolution of predator–prey interactions [5].

Prey selection is influenced by individual characteristics of predator and prey, such as sex, age, physical condition, size and behaviour. The selection pattern is also determined by the abundance and life history traits of the prey population as well as temporal and spatial activity patterns [6–9]. Characteristics of the predator that influence selection are its hunting tactic (stalking vs. coursing) and group size [10]. The interplay between predator and prey population is modulated by habitat and environmental conditions, which give either prey or predator an advantage. For example, stalking predators need cover to access prey, whereas coursing predators prefer open habitat to outrun prey [7]. In winter, deep snow cover may increase the impact of predation by reducing the mobility of ungulate prey species with a high foot load, while it affects the predator to a lesser extent. This advantage enables predators to catch even prey that are much larger than themselves (Mech et al. 2001, Hebblewhite 2005).

Predators hunting in groups are able to kill larger prey species, whereas solitary predators generally prefer species close to their own body size [11–13], possibly because of the higher risk of injury for a solitary predator attacking large prey [12]. Coursing predators probably select the slowest and weakest prey individuals, i.e., young, old or sick animals [14–16]. By contrast, stalking predators pursue their prey only over short distances, which should lead to random choice of a prey individual within a given size class [17–19]. Nevertheless selective predation by stalking predators can arise in every component of the behavioral sequence, which consists of encountering prey, the decision to make a hunting attempt on encountered prey and the actual hunting attempt [20]. Therefore not only cognitive decisions of the predator, but also prey behaviour plays an important role in the selection process.

Roe deer (*Capreolus capreolus*) is the preferred prey species of the stalking predator Eurasian lynx and when anthropogenic hunting is absent, the major causes of death are predation of fawns by red fox [21,22] and of adult animals by lynx [23,24]. A roe deer of either sex is only slightly heavier than a lynx, so lynx are able to kill individuals of all age and sex classes. Consistent with theory [19], no evidence for selection of sex or age has been found [23,25–28]. However, a precondition to determine whether there is selective predation on specific age or sex classes is a detailed knowledge of the structure of the standing prey population. This is often difficult to obtain, especially in large forested areas with low visibility. Although previous studies used the best methods available, it is widely recognized that such methods are prone to bias and imprecision [29,30]; Recently, [31] found evidence for lynx prey selection. Their results suggest that lynx select for adult roe deer in summer and winter. In summer they found a selection against yearlings and fawns were underrepresented. However, the proportion of animals that fell into the respective age and sex groups was not measured directly, but instead estimated from survival and reproduction rates. Because of the difficulties in assessing the composition of prey populations, the question whether lynx predation on roe deer is always random or is in fact selective under certain preconditions is still open.

Apart from individual traits such as sex, age and body size, prey vulnerability also depends on prey behaviour. The risk allocation hypothesis predicts how prey trade-off time spent on anti-predator behaviour with other essential activities, such as foraging and reproduction, to maximize fitness [32]. When predation risk is temporally uniform, the anti-predator behaviour of prey should vary little, whereas when predation risk greatly varies temporally, the behavioural response of prey should be strong [33]. If prey activity increases predation risk, e.g. by increasing the frequency of encountering a predator, the risk allocation hypothesis predicts that activity should be highest during a pulse of safety and lowest during a pulse of risk [34].

Felids are generally crepuscular or nocturnal [35], although the anatomical structure of their eyes makes them well suited to function under a wide range of light conditions [36]. Hunting conditions are optimal at twilight and night, with sufficient light to spot prey and with opportunities to seek camouflage for stalking. The Eurasian lynx is crepuscular, with activity peaking at dusk and dawn and lowest activity during day [37]. During periods of high predator activity, the frequency of predator-prey encounters and therefore the predation risk are expected to be highest. As a consequence, roe deer should show the strongest anti-predator response during twilight. Roe deer may adapt to the increased predation risk by decreasing their activity at twilight. This is likely to reduce the encounter rate as well as the chance of being detected.

Most studies analysing prey selection have either regarded prey of one species as identical specimens or only differentiated between sex and age classes [24,38], Considerable interest in these differences has arisen in recent years [39]. Recently it has been shown that individual variation in behavioural traits, such as habitat use and predator avoidance tactics, can entail variation in predation risk [40–42]. However, to date these individual differences in traits of ungulate species have been considered only in one study, in that of bighorn sheep (*Ovis canadensis*) [43], whose results provide evidence for a predator-induced selection favouring bold and non-docile animals. The activity patterns of roe deer show a high degree of individual variation [44], which offers the possibility of exploring whether individuals with a higher activity level during periods of high risk face a higher predation risk.

Here we tested whether the stalking predator lynx selectively preys on animals of optimal size, i.e. red deer and roe deer. We also tested whether lynx select among roe deer prey by age, sex and individual behaviour. To tackle the difficulties in assessing the composition of standing prey populations, we based our inferences on two independent data sets obtained from the same roe deer population.

## Materials and Methods

### Study area

The Bohemian Forest Ecosystem along the German–Czech border is the largest contiguous region of strictly protected woodlands in Central Europe. It represents a forested low mountain range and includes the Bavarian Forest National Park (240 km²; 49°3'19"N, 13°12'9"E) on the German side and the Šumava National Park (690 km²; 49°7'0"N, 13°36'0"E) on the Czech side. The elevation ranges from 600 m a.s.l. in the valleys to 1,456 m a.s.l. on the summits. The area is characterized by long (5–8 months), cold and snowy winters, followed by relatively warm summers. The average annual temperature lies between 6.7°C in the valleys and 3.9°C at high elevations. Annual precipitation usually ranges from 1,085 mm to 1,860 mm. In winter, the average snow depth is 40–60 cm in the valleys and 100–120 cm at higher altitudes, where maximum values of about 3 m can be reached. At higher elevations, the dominant tree species Norway spruce (*Picea abies*) is accompanied by mountain ash (*Sorbus aucuparia*); lower elevations are characterized by Norway spruce, European beech (*Fagus sylvatica*) and silver fir (*Abies alba*) [45]. Spruce trees of the national parks were massively attacked by the spruce bark beetle (*Ips typographus*). By 2013, this resulted in the death of mature spruce stands over an area amounting to 7,000 ha [46].

As in most of Europe, the Eurasian lynx became extinct in this area in the mid-19th century [47]. Its reintroduction began in Bavaria in the early 1970s, with the release of an uncertain number of animals (5 to >10, [48]). Between 1982 and 1989, 18 lynx were released on the Czech side of the Šumava Mountains [49]. At present, this lynx population is stagnant [50], with estimated densities ranging from 0.4 to 0.9 lynx/100 km² for the core area [51]. The main prey of lynx on the German side is red deer, with a density of 1.56 animals/km² estimated via coordinated counting at feeding stations in the Bavarian Forest National Park during winter. The roe deer density ranges from 1.1 to 5 animals/km² [52]. Higher densities of both deer species are found in the Czech Republic [53]. Hunting of roe deer is completely banned in both national parks; hunting in the surrounding area is restricted to specific hunting seasons and regulated by hunting plans. Red deer is hunted both inside and outside the national parks [53].

### Data preparation

#### Animal trapping and telemetry

Telemetry data were acquired between 2004 and 2012. Lynx (six males and four females) were captured using walk-through box traps either at a kill site or on known lynx trails (Table 1). All lynx were immobilized with 1–1.2 mL “Hellabrunner Mixture” (400 mg ketamine and 500 mg xylazine) and fitted with GPS mini-collars (VECTRONIC Aerospace, Germany, 350 g; see [54] for a detailed description of the handling protocol). We identified locations where lynx may have killed a prey of medium to large size as clusters of night GPS and/or VHF positions and searched for prey remains at such locations with the help of a GPS receiver and dogs. (see Podolski et al., 2013 for further details). In order to test if kills were missed using the outlined search strategy, we searched for prey remains at 300 randomly chosen GPS positions which were not part of a cluster but which were recorded by collars during the period in which lynx is generally hunting or feeding.

**Table 1:** Distribution of radio-collared animals during the study period according to sex and agegroup (juvenile or adult).

Species	Sex	Adult	Juvenile	Sum
Eurasian lynx	Females	4	0	4
	Males	6	0	6
	Sum	10	0	10
Roe deer	Females	60	26	86
	Males	55	37	92
	Sum	115	63	178
Red deer	Females	61	0	61
	Males	36	0	36
	Sum	97	0	97

Roe deer were trapped during the winter months using wooden box traps. Animals were lured into the traps with various types of bait. Captured deer were pulled from the traps by their legs; we quickly measured morphological traits and body mass and attached radio-collars. Age was determined by tooth wear. The animals were not chemically immobilized. We equipped 246 roe deer with GPS Plus collars (VECTRONIC Aerospace, Germany, 480 g; see [55] for a detailed description of the handling protocol).

Red deer were either captured within a corral or shot with an immobilization gun. The animals were attracted to the corral by food (grass silage, hay, apple pomace, sugar beets). The diameter of the corral was 20–50 m and its height was 3.5 m. Within the corral, the deer were guided to a capture facility, where the collars could be attached through hatches in the wall. This procedure did not require immobilization of the deer. Animals shot with an immobilization gun (2.5–3 mL Hellabrunner Mixture; 400 mg ketamine and 500 mg xylazine) were lured to a baiting place and shot from a raised seat. All red deer were equipped with GPS Plus collars (VECTRONIC Aerospace, Germany, 750 g; see Heurich 2011 for a detailed description of the handling protocol).

The handling protocols have been approved by Ethics Committee of the Government of Upper Bavaria and the Czech Central Commission for Animal Welfare and fulfil their ethical requirements for research on wild animals (permit number: 55.2-1-54-2532-82-10). Both mentioned institutions specifically approved this study. In addition, permits for wild animal capture were obtained from the Government of Lower Bavaria (permit number: 55.1–8621.1–57), the Czech Central Commission for Animal Welfare (permit numbers: 44048/2008-17210, 44048/2008-10001) and the Czech Ministry of Environment (permit number: 41584/ENV/10-1643/620/10-PP8). Searching for prey in the field was allowed by the Administrations of the Šumava National Park and Bavarian Forest National Park. Outside of the parks, landowner permission was obtained.

#### Camera trapping

To evaluate the age and sex class structure of roe deer and red deer populations (categories: fawn, i.e. < 1 year old; adult female; adult male), we used data from camera traps, which were in the field from November 2008 to April 2012 [51]. To ensure that the data obtained from camera traps provided a representative sample of the monitored animal populations (including both deer species), a systematic grid of 2.7 km × 2.7 km was laid across the study area, and a camera trapping site was set up in every second grid cell. We installed two opposing cameras about 70 cm above the ground on 65 sites. We used the white flash camera trap model Cuddeback Capture (Cuddeback Capture^™^ Green Bay, WI, USA), the delay between images was set at 30 s and the cameras ran 24 h per day. Sites were located on hiking trails, forest paths, roads and game paths.

Red deer and roe deer were identified on camera-trap photographs and assigned to age and sex classes whenever possible. We defined “deer years” as lasting from 1 June of each calendar year to 31 May of the following calendar year, so that the birthing periods of both species corresponded to the beginning of a new year.

#### Vehicle Collisions

We recorded all vehicle collisions with deer within the national park borders from November 2008 to April 2012 and determined species and sex.

### Data preparation and statistical analysis

Two different methods were used to analyse the different types of data: (1) data of documented prey killed by radio-collared lynx and the standing prey population according to systematic camera trapping were analysed using Manly’s standardized selection ratio alpha and (2) data on tagged roe deer were analysed using a Cox proportional hazards model with survival as the outcome variable.

#### Method 1: Comparison of prey present and prey killed

The age and sex class distribution of killed deer was obtained from kill series of radio-collared lynx. The composition of the standing prey populations was deduced from camera trap photographs. Individuals passing a camera trap may be photographed several times. To avoid counting the same individual twice, photographs taken less than 5 min apart were counted only once if the photographed individual belonged to the same species, age and sex class.

Individuals with higher movement rates are more likely to be captured by camera traps [56]. Since this may bias estimates of the population’s age and sex class structure, we restricted our analysis to periods of the year when our telemetry dataset indicated equal mobility of the two sexes. GPS position fixes were obtained by sampling from the telemetry dataset at regular intervals of either 40–80 minutes or five to seven hours in order to analyse movement patterns at two different time scales. Dividing the distance between sampled GPS position fixes by the length of the time interval and averaging yields monthly velocity values for every individual. Only monthly averages based on at least 150 (40–80 minute time interval analysis) or 30 (5–7 hour analysis) GPS position fixes of the focal individual were retained in the analysis. For every month population averages of these individual velocity values and 95% confidence intervals were plotted to discern time periods of similar mobility of the two sexes from time periods when mobility of the two sexes diverged. Except for the first weeks after birth, fawns of both species closely follow their mother [57]. Therefore, we assumed that fawns have a detection probability equal to that of their mother from September onwards. Prey preference was evaluated using Manly’s standardized selection ratio alpha [58]. For both deer species, we analysed selection between age classes and adult sex classes separately. Alpha ranges from 0 to 1; it equals 0.5 if selection is absent in our system of two prey types (juvenile versus adult, and adult female versus adult male). Ratios below the threshold indicate relative avoidance; ratios above the threshold indicate relative preference. The period of analysis was set to 2008–2012, when both camera trap and kill data were available simultaneously. Since live and killed juveniles may escape detection in early summer, selection between age classes was analysed based on kill data from the winter months (September to March for roe deer and November to May for red deer). By contrast, we used kill data from the entire year when testing whether a particular sex class was preferred among adult prey. Data from consecutive years of the study period were pooled if neither the composition of the prey population nor the distribution of kills differed significantly between years as evidenced by likelihood ratio tests. Pivotal confidence intervals for Manly’s alpha were obtained by bootstrapping [59]. For each bootstrap sample, the kill data as well as the camera trap data were randomly sampled with replacement, and Manly’s alpha was calculated. This ensures that uncertainty in the estimation of the relative use and the relative availability of prey age and sex classes were both taken into account [60]. In addition to calculating Manly’s alpha, we compared the distribution of kills with that of the prey present using likelihood ratio tests. If the composition of the prey population varied significantly between years, we inferred selective predation based on logistic regression with sex or age group as dependent variable and year and sample type (i.e. kill or camera trap event) as categorical explanatory variables. A significant non-zero effect of sample type provides evidence for selective predation. To infer if the skew in the sex ratio of the roe deer standing population was consistent across years, the proportion of males recorded by camera traps was evaluated on a yearly basis using two-sided exact binomial tests. P-values for individual years were Bonferroni-adjusted [61]. All analyses were performed in R using the packages reshape2 [62], deducer [63] and binom [64].

#### Method 2: Prey selection based on tagged roe deer

The collars were equipped with internal mortality sensors. If the animals were not active for more than 6 hours, GPS positions were realised and sent via SMS. Subsequently, the animals were located in the field, and an autopsy was performed to determine the cause of death. Roe deer collars carried dual-axis acceleration sensors (x- and y-axes) that take a measurement every 8 s and save an integrated value every 5 min. The value scale ranged from 0 (no activity) to 255 (very high activity; [65]. Since the data of the x and y-axis represents different directions, we used the sum of both axis, which is supposed to be the best representation of roe deer activity [44,66]. Utilizable activity data were retrieved for 120 roe deer. We defined movement of the roe deer body as activity because it included all possible actions (e.g. walking, running, feeding, interacting). As the roe deer body moved more, the values detected by the acceleration sensors increased. However, it was not possible to distinguish the different types of behaviour except for the distinction between activity and inactivity, which included resting and sleeping. Therefore, we defined all values from 0 to 4 as inactive behaviour according to [66]. This limit was chosen because the acceleration sensors were very sensitive to movements of the collar; they even detected light head shakings of the roe deer, which should not be considered as activity. In the next step, we split the dataset into single months and calculated the portion of active time during the activity period of lynx [37] (twilight and night) based on this threshold.

We obtained times of sunrise/sunset and nautical twilight (centre of the sun is geometrically 12 degrees below the horizon) from the US Naval Observatory [67]. As basis for this calculation, we used the coordinates 48.908 latitude and 13.381 longitude as the geographical centre of all trapping locations and home ranges.

To describe the impact of winter conditions on the mortality of roe deer, we used mean temperature and snow height from the weather station Waldhäuser (945 m NN) located in the centre of the study area.

We analysed selection by lynx according to sex, age and activity of the radio-collared roe deer using a Cox proportional hazards model with survival as the outcome variable. It describes the hazard rate by means of a linear function of fixed and time-dependent variables. To control for multicolinearity, separate models were calculated for variables with a variance inflation factor >2.5. For the proportional hazards analysis, main and two-way interaction effects of the fixed and time-dependent explanatory variables (Table 2) define the full model. The validity of the proportional hazards assumption for the variables included in the model was tested by assessing the time dependency of the regression coefficients [68,69]. Finally Wald-, score- and likelihood-ratio tests for the global null hypothesis were conducted. All analyses were performed in R [70] using the package Survival [69].

**Table 2:** Overview of fixed and time-dependent explanatory variables of the Cox proportional hazards model.

Variable	Variation
Sex	Fixed
Age	Yearly
Proportion of active time twilight	Monthly
Proportion of active time day	Monthly
Proportion of active time night	Monthly
Winter severity	Monthly

## Results

### Frequency of lynx kills

Of 394 kills found in the field prey species remained unknown in 7 cases. Of the remaining 387kills 79% were roe deer and 17% were red deer. Although only 60% of the collared lynx were males, 86% of the red deer kills were by male lynx.After excluding the 15 prey other than roe deer and red deer, we obtained a deer prey dataset of 372 lynx kills. Female and male roe deer were equally represented in lynx kills (51.3% females, 48.7% males; n = 152). This did not differ between summer (1 April to 31 October: 51% females, 49% males) and winter (1 November to 31 March: 52% females, 48% males) as well as between reproductive season (1st May to 31st August: 46% males, 54% females; n = 68) and during the remaining months of the year (1st September to 30th April: 51% males, 49% females, n = 84). Male lynx killed equal numbers of female and male roe deer (50.7% males, 49.3% females; n = 67), as did female lynx (47.1% males, 52.9%; n = 85).

The majority of roe deer kills found were adults (68.9% adults, 31.1% juveniles; n = 196). The proportion of juveniles in summer (84.1% adults, 15.9% juveniles) was lower than in winter (57.3% adults, 42.7% juveniles). The proportion of adult and juvenile roe deer killed by male lynx (71.1% adults, 28.8% juveniles; n = 90) was similar to that killed by female lynx (67.0% adults, 33.0% juveniles; n = 106).

More killed female red deer were found than killed male red deer (20.8% males, 79.2% females; n = 24). Juvenile red deer kills were found more often than adult female red deer kills (86.7% juveniles, 13.3% adults; n = 60). Adult male red deer were not among the found prey remains.

### Structure of the standing roe deer population and lynx selection

**Fig 1:**
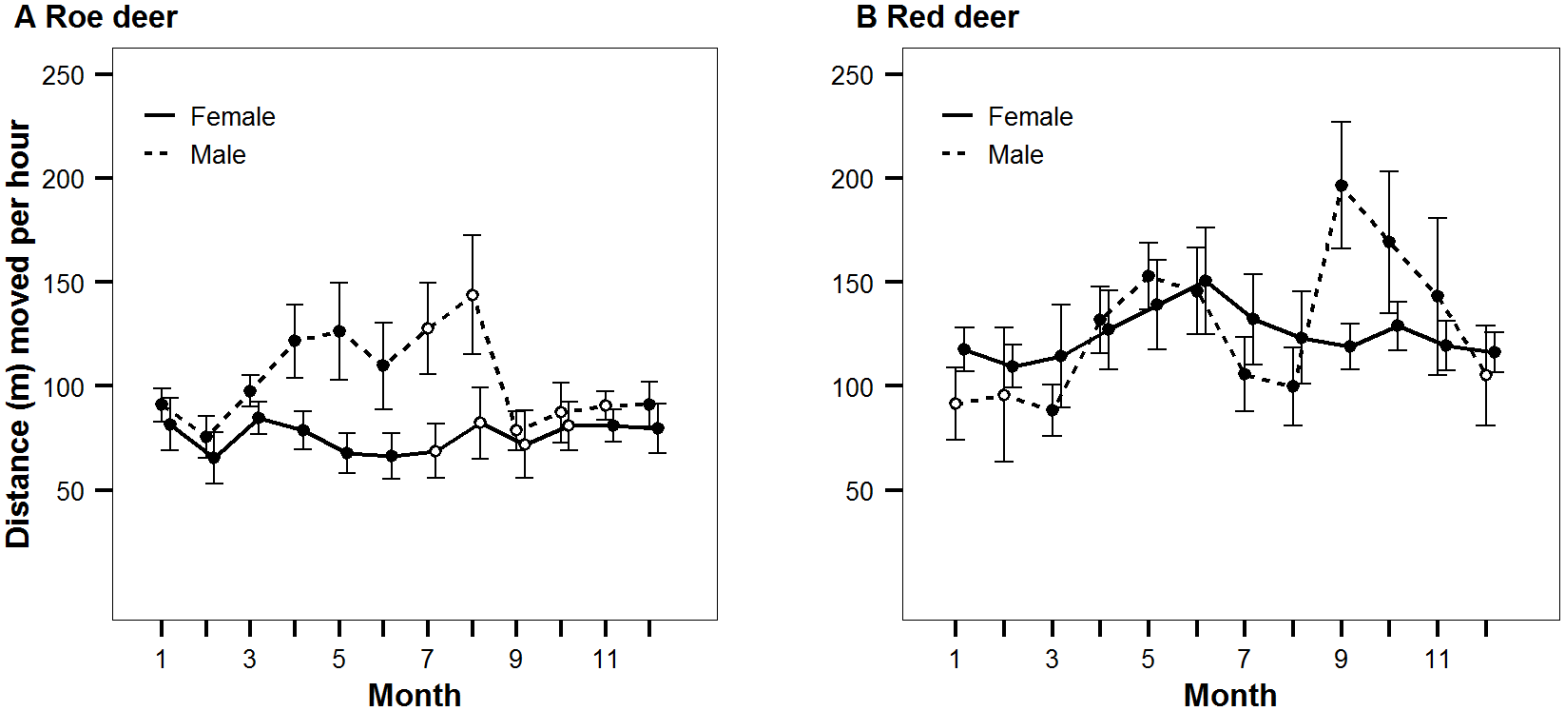
Distances covered in one hour by the two sexes in roe deer (A) and red deer (B). The interval between position fixes used for estimation ranged from 40 to 80 minutes to analyze movement patterns at a fine scale. Error bars depict the 95% confidence interval of the mean. Averages for every month and sex are based on a minimum of six individuals (solid circles: n>10, open circles: n<10).

**Fig 2:**
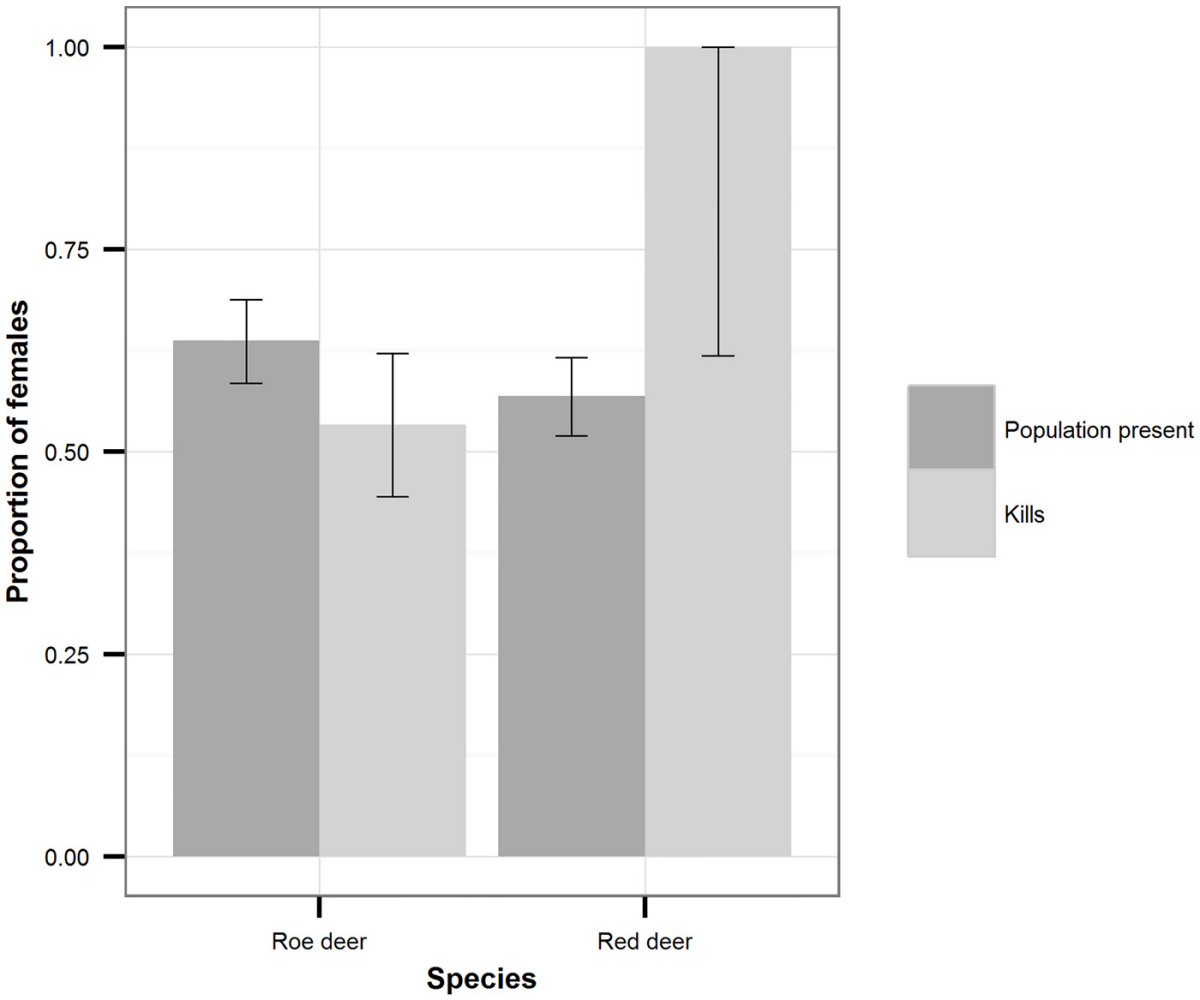
Proportion of adult females to adult males in the standing population and among kills. Data on the standing population were from camera traps; kills were those found in the field. 95% confidence intervals were calculated using the Clopper-Pearson exact method (Clopper and Pearson, 1934).

**Fig 3:**
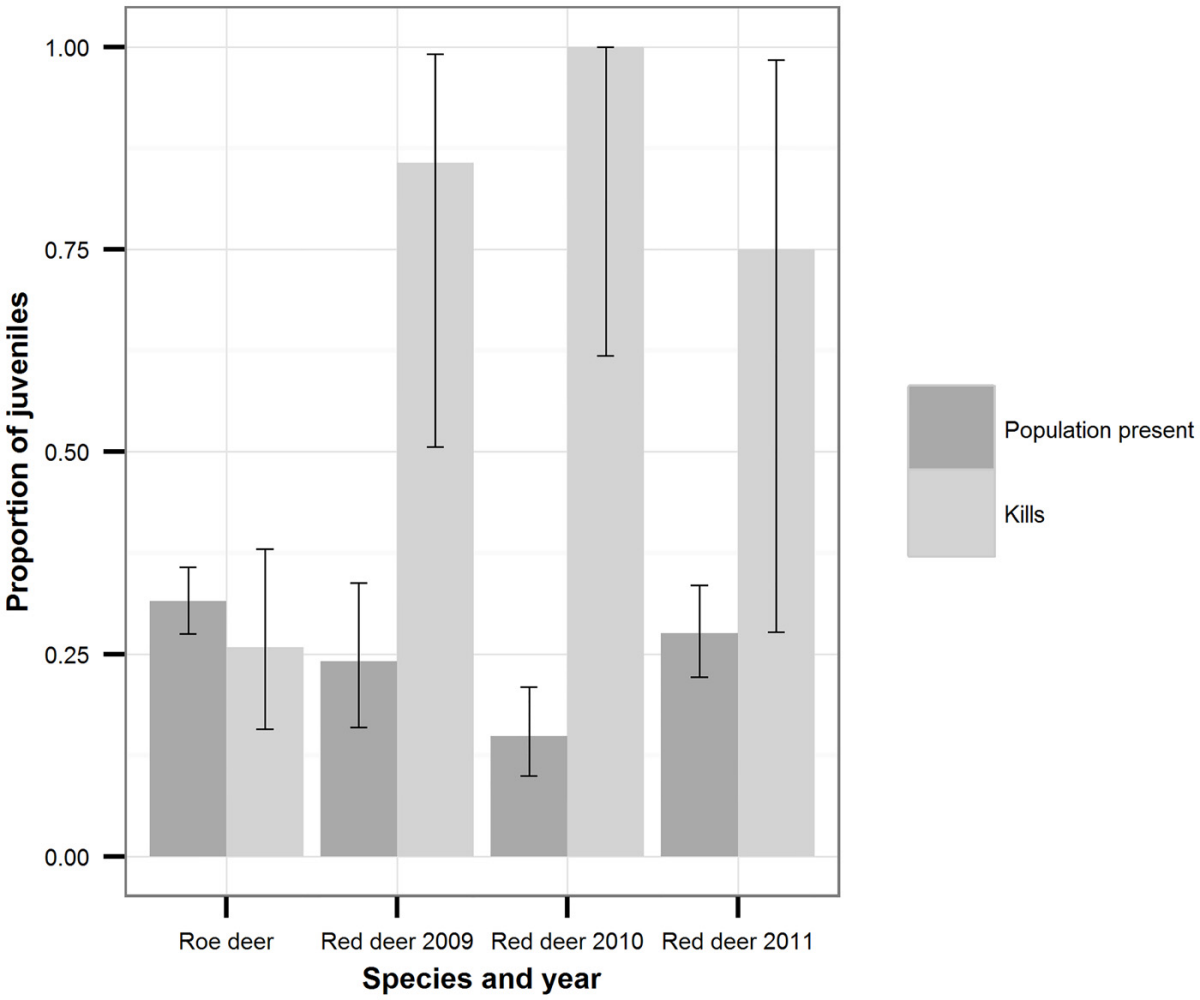
Proportion of juveniles in the standing population and among kills. Data on the standing population were from camera traps; kills were those found in the field. As the proportion of juvenile red deer in the standing population varied significantly between years, red deer data are presented separately for each year. 95% confidence intervals were calculated using the Clopper-Pearson exact method (Clopper and Pearson, 1934).

Our telemetry data provided evidence that roe deer males were more mobile than females from April to August, as would be expected since they are rutting. During the rest of the year (September to March), the distances moved per hour were equal for the two sexes (Fig 1). This held for small-scale as well as large-scale movement patterns, as evidenced by analysis of different measurement intervals (S1 Fig). Therefore, photos obtained from camera traps from September to March of the following year can be considered representative of the roe deer standing population. In all years of our study period, we obtained photos of more roe deer females than males (Fig 2). While this difference was only a trend during the two years in which we obtained few roe deer camera-trap photos (2008: n = 12; ratio of male count divided by total count m/n = 0.25; 2009: n = 58; m/n = 0.47), the difference was significant during the two years in which we obtained many photos (2010: n = 144; m/n = 0.37; p = 0.01; 2011: n = 114; m/n = 0.32; *p*<0.001; two-sided exact binomial tests with Bonferroni-adjusted p-values). Additional support for a sex bias toward females came from records of animal–vehicle collisions that occurred in a time of equal mobility (September to March; *p* = 0.023, n = 24; two-sided exact binomial test). From September to March, approximately one-third of the roe deer population was composed of fawns (Fig 3). Neither the sex ratio of adults nor the proportion of fawns varied significantly between years (adult sex ratio: *G* = 4.4, *df* = 4, *p* = 0.35; proportion of fawns: *G* = 7.9, *df* = 4, *p* = 0.10). We therefore pooled data from all years for the analysis of prey selection.

The adult sex ratio in the roe deer kill data was significantly different from that of the standing roe deer population according to camera trap data (*G* = 4.0, *df* = 1, *p* = 0.046; Fig 2). Lynx preyed significantly more on male than on female roe deer (Manly’s alpha = 0.61, bootstrapped 95% confidence interval = 0.51–0.71). From September to March, the proportion of juvenile kills did not differ significantly from the proportion of juveniles in the standing population (Manly’s alpha = 0.43, bootstrapped 95% confidence interval = 0.29–0.58; Fig 3).

### Structure of the standing red deer population and lynx selection

Our analysis of GPS data of red deer indicated similar movement rates of the two sexes from November to May (Fig 1). We therefore evaluated the composition of the red deer standing population using camera trap photos only from this time period.

Adult females were captured by camera traps more frequently than males in all years except 2008, when sample size was lowest When data from all years were pooled, the sex ratio was significantly skewed toward females (n = 401, p < 0.001; two-sided exact binomial test; Fig 2). Juveniles constituted slightly less than one-third of the red deer standing population, but as the exact proportion varied significantly between years (*G* = 9.8, *df* = 3, *p* = 0.02; Fig 3), data from different years had to be considered separately.

Our logistic regression model investigating selective predation by lynx on juvenile and adult red deer showed that calves were significantly overrepresented in lynx kills but not in the standing population (Table 3). Only four adult red deer kills were found in the field, all of which were female. Likely due to this small number of adult red deer killed by lynx, the sex ratio obtained from lynx kills did not differ significantly from that from camera-trap events (*p* = 0.14; Fisher’s exact test).

**Table 3:** Output of the logistic regression model used to infer selective predation on juveniles over adult red deer. S.E., standard error.

Variable	Estimate	± S.E.	Δdf	D	p-value
Year 2008	−0.955	0.372	3	8.887	0.031
Year 2009	*−*1.156	0.248			
Year 2010	*−*1.705	0.217			
Year 2011	*−*0.976	0.145			
Sample (kill vs. camera trap)	3.156	0.776	1	26.843	<0.0001

### Prey selection based on radio-collared roe deer data

Within the study period, 93 (77.5%) of the radio-collared roe deer died during the lifetime of the collar. Causes of death were lynx predation (46 cases), hunting (23), vehicle collisions (9) and other causes (7).

According to the results of the Cox proportional hazards model with fixed and time-dependent variables, lynx selected male roe deer over females. A male roe deer had a 1.8-fold greater chance of being killed than a female roe deer (Table 4). In addition, lynx selected roe deer with low activity levels. For every 10% decrease in the proportion of active time at twilight, the probability of being killed increased 1,39-fold. Moreover the interaction between snow depth and temperature had a significant effect. Higher snow depths in combination with low temperatures, decrease the survival of roe deer.

**Table 4:** Output of the Cox proportional hazards model with fixed and time-dependent variables.

Variable	Coefficient	Exp (coefficient)	Pr(>|z|)
**Age**	0.006547	0.006712	0.33
**Sex female**	-0.587060	0.555959	0.07
**Activity twilight**	-0.0328683	0.9676660	0.04
**Activity night**	-0.023329	0.976941	0.31
**Snow height**	-0.002521	0.997483	0.64
**Temperature**	-0.005378	0.994637	0.90
Snow height *Temperature	-0.001723	0.998278	0.03

## Discussion

In this study, we analysed lynx selection of red deer and roe deer prey using two independent data sets obtained from the same populations. One data set contained data on the number of ungulates killed by radio-collared lynx and the prey population structure based on systematic camera trappings. In red deer, which is the prey species with the largest body size, lynx preferentially preyed on calves and females and selected against males. In roe deer, whose body size is approximately equal to that of lynx, lynx preyed preferentially on males. Prey individuals that were inactive during high risk periods were at a greater risk of lynx attack.

Previous studies have shown that where lynx lives sympatrically with roe deer, lynx selects for this prey species [23,25]. The results of our study are in line with these findings as roe deer was killed more often than red deer. This selection of roe deer over red deer has been attributed to the similar sizes of roe deer and lynx and occurrence of roe deer in small groups in habitats that provide sufficient cover, in contrast to the much higher weight of red deer and their living in larger groups that prefer open areas that make it more difficult for lynx to stalk them [19,31,71].

The body size sexual dimorphism of roe deer is very low [72]; the difference in body mass between sexes is below 5% [73] and there is no age-related increase in body size in individuals older than two years [74]. In the absence of a prolonged pursuit, lynx and other stalking predators kill their prey within 20 m with a success rate of more than 70% [75,76]. As a consequence, it is believed that lynx choose individuals that can be captured most efficiently with minimal risk [10]. For prey species lacking body size sexual dimorphism, this should lead to random selection [7,17,19], as has been observed in earlier studies of the lynx–roe deer predator–prey relationship (Andersen et al., 2007; Okarma et al., 1997; Jedrzejewski et al., 1993). Our results, by contrast, indicate that lynx select male roe deer over females. As the small differences in body size cannot explain this selection, we assume that behavioural differences predispose males to higher rates of predation. Roe deer males spend more time alone than females, which form small groups with their offspring [77]. Thereby males are supposed to have lower chances of detecting the predator before it attacks [78,79]. In addition roe deer males seem to bebe bolder than females and more aggressive among each other [80]. These behavioural differences might make them more susceptible to lynx predation then females. Surprisingly, found no increase in the proportion of male kills among total kills during the summer months, when the males are defending territories, while the females are more secretive raising their fawns. Similar to our findings for Eurasian lynx, cheetahs (*Acinonyx jubatus*) select for males when preying on Thomson’s gazelles (*Gazella thomsonii)*, despite their low body size sexual dimorphism [81]. This prey selection pattern is also driven by prey behaviour: Cheetahs preferentially kill individuals at the edge of the group, which are mostly males [81]. Behaviour also influences vulnerability of bighorn sheep (*Ovis canadensis*) and wapiti (*Cervus canadensis*) to predation. Old female bighorn sheep travel ahead of the group and are thus more vulnerable to ambush by pumas [82]. Wapiti bulls show less anti-predator behaviour than cows to wolves, and therefore face a greater risk of predation [83].

In our study, we found no evidence for selection of adult roe deer over juveniles by either male or female lynx. Our results are in accordance with the results of other studies that show that lynx do not select for roe deer fawns (Okarma et al. 1997, Molinari-Jobin et al. 2002, Mejlgaard et al. 2013). Roe deer fawns of 2.5–3 months follow their mothers [84] and their activity patterns are synchronized [44], which should make them as vulnerable to attack as females in this respect. However, a fawn is weaker and slower than an adult, which reduces its chance of outrunning a lynx. This disadvantage may outweigh the factors causing the predator to select adult animals. Mixed results have been obtained on the prey selection of pumas: While two studies concluded that pumas select for fawns of white-tailed deer (*Odocoileus virginianus*) and mule deer (*Odocoileus hemionus*) [18] [85], others found selection for adult mule deer [7]. Our results show that lynx kill a significant proportion of adults, in particular prime-age animals, which can have a strong impact on recruitment and population size of the prey population [86]. However, the observed selection for males is expected to reduce this impact [3].

Our results of lynx selection of red deer differ from the roe deer results. Lynx select for red deer calves and females. So far, lynx selection of red deer has only been investigated in the Bialowieza primeval forest. Our results on selection in red deer are in accordance with findings of these earlier studies [23,25]. Most red deer in our study were killed by male lynx, which are 15–20% heavier than females in the study area. This follows theoretical expectations that the most profitable prey type of a large predator should be the largest available prey that can be killed safely [87,88]. The larger body size of red deer is most likely the reason that makes adult females less suitable prey than calves. Adult males, which are about five times the weight of a male lynx, are believed to be too dangerous as prey [25].

Because of the difficulties in collecting information on prey population structure, some earlier studies only describe the age and sex structures of lynx kills [27] or assume that harvests by human hunters represent the structure of the population present [26,28,89], which may be incorrect because human hunters select for e.g. males [90]. Other studies have relied on estimation of the age and sex structures of standing prey populations derived from drive census data (Okarma et al., 1997; Jedrzejewski et al., 1993) or direct observation at deer winter-feeding sites (Andersen et al., 2007). These types of data have to be interpreted with caution. In fact, there is a consensus that roe deer populations cannot be counted accurately using standard methods [29,30] and even drive counts and counting at feeding stations can be prone to bias. In drive counts in Poland (Okarma et al., 1997; Jedrzejewski et al., 1993), people in a row at least 50 m apart walk through a forest compartment. All animals that leave that compartment are counted by people surrounding it. However, roe deer are able to bypass people walking through the forest, which might affect the results of drive counts. Counts at feeding stations miss individuals that do not visit these places regularly. Estimation of population structures based on radio-collared animals [31] can be influenced by the success of trapping different sex and age classes. With all of these methods, the boldest and most competitive individuals, which are commonly males, may be overrepresented [80]. An overrepresentation of males in counts leads to an underestimation of the degree of lynx selectivity for roe deer males. One method to overcome these problems would be to use Capture-Mark-Recapture (CMR) methods.

The camera traps used in this study cover the study area uniformly and were placed on all types of trails and paths. Thus, we do not expect bias derived from over- or under-representation of certain portions of the monitored area. We also limited our analyses of camera trapping data to the periods when male and female roe deer showed the same levels of mobility; thus, an over-representation of the more mobile roe deer sex class is unlikely. Furthermore, we analysed a large group of radio-collared animals with known fate, which gives a reliable measure of prey selectivity of lynx independent of the need to measure the standing population, and the results of this second analysis supported our findings based on camera-trap data. Additional evidence for a sex ratio skewed towards females among adult roe deer comes from records of animal–vehicle collisions.

The influence of personality on prey selection has not yet been investigated much (Réale and Festa-Bianchet 2003). Here, we analysed the general activity level of roe deer, which is regarded as one category of an animal’s temperament [39]. We found that individual radio-collared roe deer differed in activity as already found by [44]. Our results showed that the individual activity behaviour also determines the predation risk, with those animals more active during twilight having a lower risk of being killed.

To successfully hunt deer, lynx needs two preconditions: first, it has to encounter deer even in areas of low deer densities, and second, it needs to remain undetected after the encounter while it stalks its prey. An actively moving deer is easier to detect because a lynx relies mostly on its visual sense for hunting. On the other hand, after the predator has detected its prey, it should be easier for it to stalk a resting deer than an active, more vigilant one. Low activity as measured in this study is connected with sleeping, resting and chewing. In this position, deer have a poor view of the area and might not detect the predator early enough to escape an attack, which is likely one reason why roe deer choose open habitats with good views when inactive in the study area [91], while selecting sheltered habitat in Southern Norway, where no lynx are present [92] In summary, being active likely increases the risk of being detected by a lynx, but it may also increase the chance of detecting the lynx before it attacks. To reduce the risk of detection, roe deer are usually active in habitats with a dense understory [91], where lynx cannot easily spot them. In addition, the probability of escaping an attack is higher when the prey is active and not lying on the ground. Being more active during twilight, when also lynx is most active [37], might thus be an adaptation to reduce the chance of being killed during a period of high predation risk. Our findings are in accordance with the risk allocation hypothesis, which predicts the strongest behavioural response during periods of pulsed risk, which can be found in our system during twilight. Our findings are also in keeping with the results of [89], who showed that roe deer are killed mainly while ruminating, i.e. during the inactive period. During rumination, the prey’s sense of hearing may be greatly impaired and at the same time, visual senses cannot be used effectively when the animal is lying close to the ground.

At night, roe deer activity was generally lower, and higher individual activity had no influence on the survival of the animals. In the dark, roe deer rely more on their hearing and smell than on their visual sense [93]; Therefore, being active might not be advantageous because movement makes noise, which may prevent the prey from hearing an approaching predator and may also attract the predator. This suggests that increased activity offers an advantage only during dusk, when the light level is sufficient for visual scanning of the surroundings. This interpretation follows the results of Eckard et al (2015), who found a lower vigilance level of roe deer at night than during the day in the same study area, where lynx was present.

Climatic factors, especially winter conditions, can have a major influence on prey vulnerability. High snow packs limit the access to food and increase the energy needed for movement and thermoregulation [94–96]. In line with previous results [24,97], we found that higher snow depths in combination with low temperatures decrease the survival of roe deer.

Felid predators living in northern environments, such as Canadian lynx and Eurasian lynx, are well adapted to high snow cover by having large paws, which can serve as snowshoes [35,98], giving them an advantage over ungulate prey, which have a higher foot load. High snow cover likely also increases the vulnerability of red deer to lynx attacks, as animals in deeper snow are more prone to predation than animals which stay closer to feeding stations were snow is denser because of trampling [99].

Our results suggest that hunting technique and prey size appear to be only two of several factors determining prey vulnerability. It seems that prey behaviour can be a major driver of predation risk in the lynx–roe deer relationship. More important than size differences are the probability of an encounter, which can be reduced by a higher investment in anti-predator behaviour [100,101].

## Supporting Information

S1 Fig Distances covered in one hour by the two sexes in roe deer and red deer. (TIF)

S1 Dataset camera_trap_events_roe_red. (CSV)

S2 Dataset distance_moved_red_deer_5to7hours. (CSV)

S3 Dataset distance_moved_red_deer_40to80mins. (CSV)

S4 Dataset distance_moved_roe_deer_5to7hours. (CSV)

S5 Dataset distance_moved_roe_deer_40to80mins. (CSV)

S6 Dataset kills. (CSV)

S7 Dataset roe_deer. (CSV)
