# Supplementary figures and images for: Selective Predation of a Stalking Predator on Ungulate Prey
Source: PLoS One. 2016 Aug 22;11(8):e0158449. doi: 10.1371/journal.pone.0158449 (PMC4993363; doi:10.1371/journal.pone.0158449)

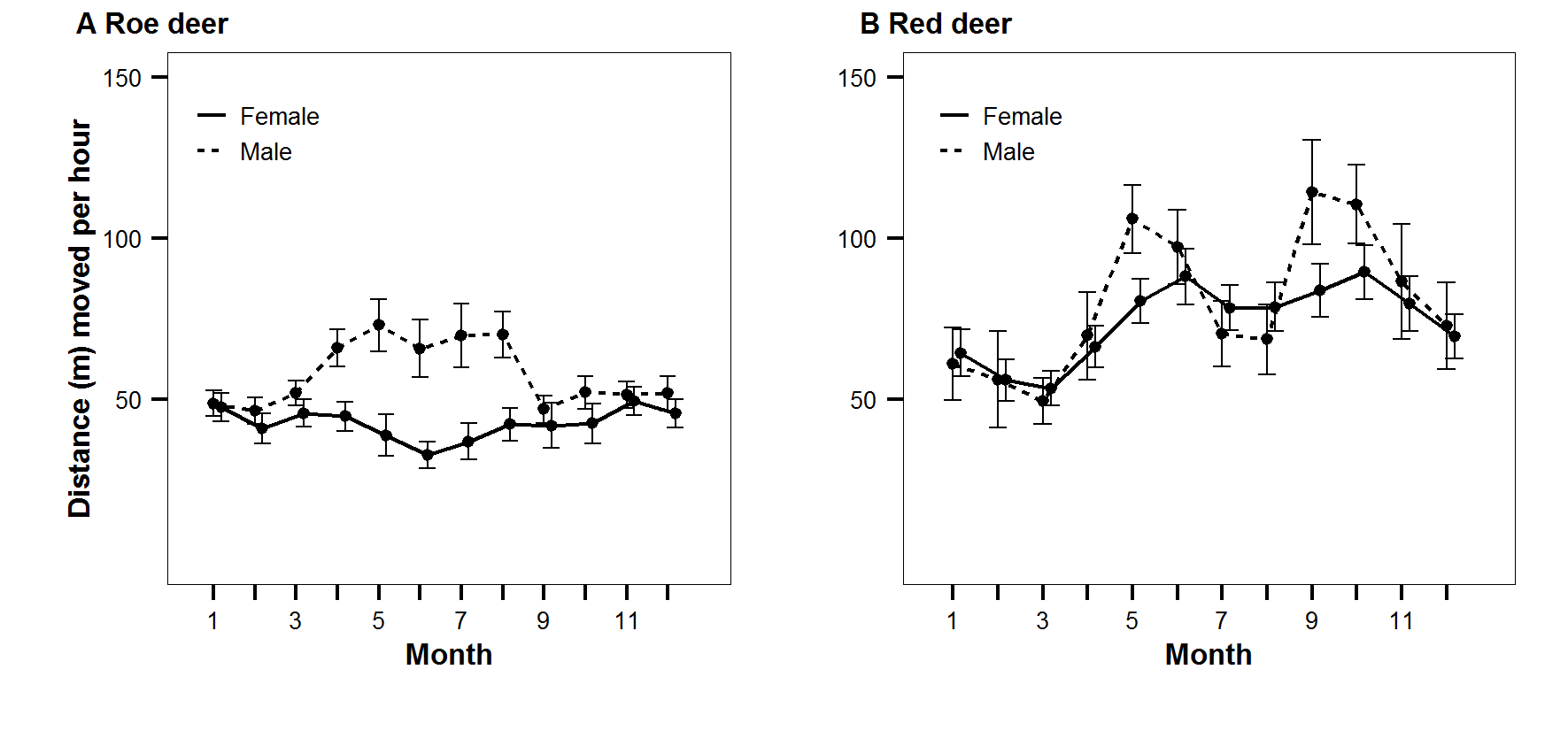

Supplement: S1 Fig — (TIF) [file pone.0158449.s001.tif]
